# New Measles Virus Genotype Associated with Outbreak, China

**DOI:** 10.3201/eid1606.100089

**Published:** 2010-06

**Authors:** Yan Zhang, Zhengrong Ding, Huiling Wang, Liqun Li, Yankun Pang, Kevin E. Brown, Songtao Xu, Zhen Zhu, Paul A. Rota, David Featherstone, Wenbo Xu

**Affiliations:** National Institute for Viral Disease Control and Prevention, Beijing, People’s Republic of China (Y. Zhang, H. Wang, S. Xu, Z. Zhu, W. Xu); Yunnan Center for Disease Control and Prevention, Kunming, People’s Republic of China (Z. Ding, L. Li, Y. Pang); Capital Medical University, Beijing (H. Wang); Beijing Children’s Hospital, Beijing (H. Wang); Health Protection Agency, London, UK (K.E. Brown); Centers for Disease Control and Prevention, Atlanta, Georgia, USA (P.A. Rota); World Health Organization, Geneva, Switzerland (D. Featherstone); 1 These authors contributed equally to this study.

**Keywords:** Measles virus, new genotype, phylogenetic analysis, viruses, research

## Abstract

To determine the origin of the virus associated with a measles outbreak in Menglian County, Yunnan Province, People’s Republic of China, in 2009, we conducted genetic analyses. Phylogenetic analyses based on nucleoprotein (N) and hemagglutinin (H) gene sequences showed that these Menglian viruses were not closely related to sequences of any World Health Organization (WHO) reference strains representing the 23 currently recognized genotypes. The minimum nucleotide divergence between the Menglian viruses and the most closely related reference strain, genotype D7, was 3.3% for the N gene and 3.0% for the H gene. A search of the databases of GenBank, WHO, and the Health Protection Agency Measles Nucleotide Surveillance showed that the Menglian viruses, together with the 2 older non-Menglian viruses, could be members of a new proposed measles genotype, d11. The new genotype designation will allow for better description of measles transmission patterns, especially in the Southeast Asian and Western Pacific regions.

## Abstract 2

Better understanding of transmission patterns will enhance control and elimination programs.

Measles virus is a negative-sense, single-stranded RNA virus in the family *Paramyxoviridae,* genus *Morbillivirus*. Infection with this virus typically causes high fever, maculopapular rash, conjunctivitis, cough, and coryza (*1*). Measles virus is monotypic, but genetic variation in the hemagglutinin (H) and nucleoprotein (N) genes can be analyzed by molecular epidemiologic techniques and used to study virus transmission patterns (*2*–*4*). The World Health Organization (WHO) currently recognizes 23 genotypes of measles virus and has established guidelines for the designation of new genotypes (*3*–*7*).

Although deaths attributed to measles have declined by 78% worldwide, from ≈733,000 deaths in 2000 to ≈164,000 in 2008, risk for illness and death from measles still exists in countries with poor routine vaccination coverage, and outbreaks are a threat in most of these countries (*6**,**8*). When the Universal Childhood Immunization goals were attained in the 1990s, illness and death from measles in the People’s Republic of China decreased dramatically. During 1995–2009, measles incidence was 5–10/100,000 population, and <250 measles-associated deaths were reported each year (*9**–**11*; National Notifiable Disease Reporting System of China [http://202.106.123.35/]). Outbreaks of measles continued to occur because of increased numbers of susceptible children, especially in areas with low routine vaccination coverage. At the end of 2009, a measles outbreak in Menglian County was reported to the National Measles Laboratory of the Chinese Center for Disease Control and Prevention. Menglian County is situated on the western side of Yunnan Province and shares a border with Myanmar. As part of routine surveillance activities, the measles outbreak was investigated. Cases were confirmed through detection of immunoglobulin (Ig) M, and virus isolates were obtained for genetic analysis.

## Materials and Methods

### Specimen Collection

Staff members from the Yunnan Center for Disease Control and Prevention collected serum and throat swab or urine specimens from patients during the outbreak (14 from Myanmar and 16 from Menglian). Serum samples were collected from 9 Myanmar and 7 Menglian case-patients. Throat swabs or urine specimens, from the first 6 Menglian patients, were obtained according to the WHO procedures for laboratory diagnoses of measles and rubella virus infections (*12*) and transported to the National Measles Laboratory for processing by standard procedures. To confirm all suspected cases, we used ELISA kits (Virion/Serion GmbH, Würzburg, Germany) to detect measles and rubella virus IgM.

### Virus Isolation, PCR, and Sequencing

Measles virus was isolated by using the Vero/hSLAM cell line (*13*), and infected cells were harvested when >75% of the culture showed cytopathic effect (*12*). Meanwhile, RNA was extracted from all clinical specimens by using the QIAamp Viral RNA Mini Kit (QIAGEN, Beijing, China) according to manufacturer’s instructions. Reverse transcription–PCR (RT-PCR) was used to amplify either the 550 nt coding for the COOH terminus of the N gene or the full-length open reading frame for the H gene (*14*). PCR products were purified by using a QIAquick Gel Extraction kit (QIAGEN). Sequences of the amplicons were obtained by using BigDye terminator version 2.0 chemistry according to the manufacturer’s protocol for both sense and antisense strands on an automated ABI PRISMTM 3100 DNA Sequencer (PerkinElmer, Beijing, China). Sequences were analyzed by using SequencerTM (Gene Codes Corporation, Ann Arbor, MI, USA) and version 7.0 of BioEdit (www.mbio.ncsu.edu/BioEdit/BioEdit.html). Phylogenetic analyses were performed and trees were generated by using MEGA4 (www.megasoftware.net). The robustness of the groupings was assessed by using bootstrap resampling of 1,000 replicates.

## Results

During 2008–2009, routine measles vaccine coverage in Menglian County was as high as 95%; the last local measles case before this outbreak had been reported in June 2008. The outbreak began when 14 persons from Myanmar became ill with fever and rash and sought healthcare in Menglian during October 10–November 28, 2009. The first measles case in a person from Menglian occurred on November 5, 2009; the last occurred on December 20. On December 28, the number of measles cases from the outbreak totaled 14 persons from Myanmar and 16 from Menglian. Measles was confirmed by laboratory detection of IgM in all 16 patients from whom serum samples were available (9 from Myanmar and 7 from Menglian). No IgM against rubella virus was detected (Table 1).

**Table 1:** Characteristics of patients with laboratory-confirmed measles, 2009*

Patient no.	Patient age	Residence†	Date of most recent vaccination	Date of disease onset
1	9 y	Myanmar	Unknown	Nov 4
2	9 y	Myanmar	Unknown	Nov 4
3	8 y	Myanmar	Unknown	Nov 4
4	7 y	Myanmar	Unknown	Nov 6
5	2 y	Myanmar	Unknown	Nov 3
6	2 y	Menglian	Unknown	Nov 9
7	39 y	Menglian	Unknown	Nov 9
8	21 y	Menglian	Unknown	Nov 5
9	18 mo	Myanmar	Unknown	Nov 13
10	6 mo	Menglian	No vaccination	Nov 14
11	13 y	Myanmar	Unknown	Nov 6
12	8 y	Myanmar	No vaccination	Nov 22
13	20 y	Myanmar	No vaccination	Nov 20
14	2 y	Menglian	2008 Jan15	Nov 19
15	5 y	Menglian	2006 Jun 7	Dec 9
16	5 y	Menglian	2008 Oct 27	Nov 5

*For all patients, immunoglobulin M against measles was positive and against rubella virus was negative. †Menglian, Menglian County, Yunnan Province, People’s Republic of China.

**Figure 1:**
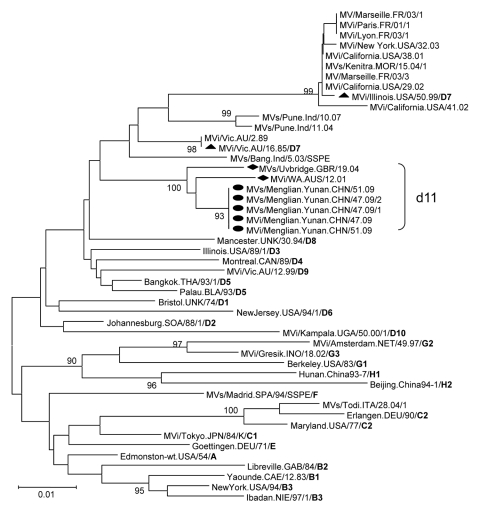
Phylogenetic analysis of the sequences of the nucleoprotein genes (450 nt) of the strains of measles virus from Menglian County, Yunnan Province, People’s Republic of China. The unrooted tree shows sequences from Menglian viruses (circles) compared with World Health Organization (WHO) reference strains for each genotype. Triangles indicate D7 WHO reference strains; diamonds, the 2 older non-Menglian strains. Genotype designation is in **boldface.** MV, measles virus; MVi, measles virus sequence from isolates; MVs, measles virus sequence from clinical specimens. Scale bar indicates base substitutions per site.

**Figure 2:**
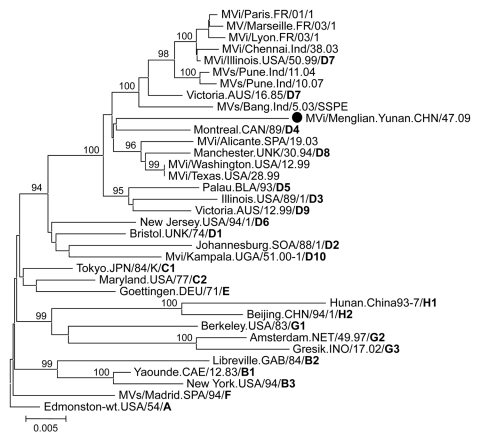
Phylogenetic analysis of the sequences of the hemagglutinin genes of the strains of measles virus from Menglian County, Yunnan Province, People’s Republic of China. The unrooted tree shows sequences from the Menglian viruses (circles) compared with World Health Organization reference strains for each genotype. Genotype designation is in **boldface.** MVi, measles virus sequence from isolates; MV, measles virus; MVs, measles virus sequence from clinical specimens; wt, wild type. Scale bar indicates base substitutions per site.

Two measles virus isolates, MVi/Menglian.Yunnan.CHN/47.09 and MVi/Menglian.Yunnan.CHN/51.09, were obtained from urine and throat swab specimens of 2 persons from Menglian. Positive RT-PCR products of the partial N gene were obtained from clinical specimens from 5 of 6 case-patients, and the entire H gene was amplified from the 2 isolates (Table 2). The sequence of the 450 nucleotides coding for the 150 amino acids at the COOH terminus of the N gene was obtained for all 5 case-patients, and the entire coding region of the H gene was sequenced for 1 representative isolate. Nucleotide sequence data for the strains from the Menglian case-patients were deposited in GenBank under accession nos. GU440571–GU440576. Sequence analysis of the C-terminal 450 nucleotides of the N gene of 2 isolates and 5 clinical specimens revealed identical sequences, suggesting a single chain of transmission. Phylogenetic and genetic distance analyses based on both N and H gene sequences showed that these viruses from Menglian case-patients were members of clade D (Figures 1, 2; Table 3).

**Table 2:** Description of measles viruses, proposed genotype d11, detected in Menglian County, Yunnan Province, People’s Republic of China, 2009*

Patient no.	Patient age	Result, by test		Strain name	GenBank accession no.
		RT-PCR	Virus isolation		
YN09–1	6 mo	+	+	MVi/Menglian.Yunnan.CHN/47.09	GU440571,† GU440576‡
YN09–2	45 y	+	–	MVs/Menglian.Yunnan.CHN/47.09/1	GU440572†
YN09–3	21 y	+	–	MVs/Menglian.Yunnan.CHN/47.09/2	GU440573†
YN09–4	6 mo	+	+	MVi/Menglian.Yunnan.CHN/51.09	GU440574†
YN09–5	5 y	+	–	MVs/Menglian.Yunnan.CHN/51.09	GU440575†

*RT-PCR, reverse transcription–PCR; MVi, measles virus sequence from isolates; MVs, measles virus sequence from clinical specimens. †Sequence of N partial gene. ‡Sequence of H gene.

**Table 3:** Genetic distances between proposed reference strain for genotype d11 (MVi/Menglian.Yunnan.CHN/47.09) and reference strains

WHO reference strain, genotype: virus name	Genotype d11, % nucleotide divergence	
	Nucleoprotein gene	Hemagglutinin gene
A: Edmonston-wt.USA/54	5.8	3.7
B1:Yaounde.CAE/12.83	7.3	4.7
B2: Libreville.GAB/84	7.8	5.6
B3: New York.USA/94	7.1	5.2
C1: Tokyo.JPN/84/K	6.0	3.9
C2: Maryland.USA/77	7.8	4.4
D1: Bristol.UNK/74	4.4	4.0
D2: Johannesburg.SOA/88/1	4.4	4.6
D3: Illinois.USA/89/1	4.2	3.7
D4: Montreal.CAN/89	4.7	3.0
D5: Palau.BLA/93	3.8	3.5
D6: New Jersey.USA/94/1	5.8	4.2
D7: Victoria.AUS/16.85	3.3	3.0
D8: Manchester.UNK/30.94	4.4	3.0
D9: Victoria.AUS/12.99	4.9	3.5
D10: Kampala.UGA/51.00/1	7.3	4.3
E: Goettingen.DEU/71	6.4	4.5
F: MVs/Madrid.SPA/94 SSPE	6.4	4.4
G1: Berkeley.USA/83	8.0	5.2
G2: Amsterdam.NET/49.97	8.7	5.8
G3: Gresik.INO/17.02	7.6	6.3
H1: Hunan.CHN/93/7	7.6	6.6
H2: Beijing.CHN/94/1	9.1	5.7

*WHO, World Health Organization; CAE, Cameroon; GAB, Gabon; JPN, Japan; UNK, United Kingdom; SOA, South Africa; CAN, Canada; AUS, Australia; UGA, Uganda; DEU, Germany; SPA, Spain; NET, the Netherlands; INO, Indonesia; CHN, China.

The sequences of these viruses were not closely related to the sequences of any of the WHO reference strains that represented the 23 currently recognized genotypes. These sequences were closest to the sequence of the genotype D7 reference strain and the contemporary genotype D7 strains available from GenBank. The minimum nucleotide divergence between the Menglian viruses and the D7 WHO reference strain (Victoria.AUS/16.85) was 3.3% for the N gene and 3.0% for the H gene (Table 3). Bootstrap analysis of Menglian H-gene sequences and the WHO reference sequences showed 100% confidence in the group containing the Menglian viruses. When the N-gene sequences were compared with WHO reference sequences and contemporary genotype D7 sequences, bootstrap support for the Menglian branch was 100% (Figure 1).

A search of the databases of GenBank, WHO, and the Health Protection Agency Measles Nucleotide Surveillance (www.who-measles.org) identified only 2 closely related sequences. The most closely related sequence was from a measles virus isolate, MVi/WA.AUS/12.01 (GenBank accession no. AF481492), previously designated as genotype D7, which had been imported from Myanmar into Australia in 2001 (*15*). The second, from a clinical specimen, MVs/Uvbridge.GBR/19.04 (GenBank accession no. GU937234), was detected in an oral fluid sample collected in 2004 from a person who had recently returned to the United Kingdom from Bangladesh. Both of these related sequences shared 98.4% nucleotide homology with the Menglian virus over the 450 nucleotides coding for the 150 amino acids at the COOH terminus of the N gene. Phylogenetic analysis showed that the 2 older non-Menglian strains (MVi/WA.AUS/12.01 and MVs/Uvbridge.GBR/19.04) formed a unique cluster combined with 5 Menglian strains (Figure 1).

## Discussion

The Menglian measles viruses, together with the 2 closely related viruses, MVi/WA.AUS/12.01 and MVs/Uvbridge.GBR/19.04, should be considered as members of a newly proposed measles genotype, d11. MVi/Menglian.Yunnan.CHN/47.09 (GenBank accession nos.: N, GU440571; H, GU440576) was chosen as the reference strain because an isolate is available, it grows to high titer in cell culture, and the sequence is representative of the Menglian strains. Although designation as a genotype is not permanent until after the new genotype has been acknowledged and formally designated by WHO, the percentage sequence divergence between the N and H gene sequences of the Menglian isolates and the sequences of the reference strains exceed the recommended threshold for designation of a new measles genotype, 2.5% and 2.0%, respectively (*3*–*5*). Phylogenetic analyses indicated that these viruses form a unique cluster compared with all previously characterized wild-type measles viruses. Because the Australia virus was imported from Myanmar and the sequence from the United Kingdom originated in a country adjacent to Myanmar, these sequences should be included as members of the proposed d11 genotype.

The most useful criterion for a new genotype is that it must have some epidemiologic utility for describing the transmission pathways of measles virus. The Menglian viruses responsible for the measles outbreak in China represent strains that are probably associated with endemic transmission of virus in Myanmar. When we consider the sequence of the Australia virus, we believe that the genotype d11 measles virus might have been circulating in Myanmar since 2001. The d11 sequences are easily distinguished from the sequences of viruses in other genotypes (e.g., D8, D4, H1, D5, D9, G3) that are circulating in neighboring countries. Therefore, the Menglian viruses are providing baseline genetic information about viruses endemic to Myanmar and possibly neighboring countries. The new genotype designation will enable better description of measles transmission patterns, especially in the Southeast Asian and Western Pacific regions of WHO.

Molecular epidemiologic data, when analyzed in conjunction with standard epidemiologic data, can help document viral transmission pathways, identify whether a virus is endemic or imported, and aid in case classification, thus enhancing control and elimination programs (*16*–*20*). Genetic characterization of measles viruses circulating in China from 1993 through 2009 demonstrated that genotype H1 was widely distributed throughout the country and that China has a single, endemic genotype (*9**–**11*; National Measles Laboratory database, unpub. data). However, data on the circulating genotypes in some other countries in the Southeast Asian and Western Pacific regions of WHO are limited. Further sequence analysis of measles virus strains circulating in Myanmar and neighboring countries should clarify the distribution pattern of this newly recognized genotype and may enable recognition of other new genotypes. Countries in the Western Pacific Region, including China, have committed to 2012 as the target year for measles elimination (*21*). However, rapidly identifying imported measles cases and controlling spread are major challenges for achieving this goal. Enhancing measles virus surveillance to quickly identify imported cases of measles will become more critical as measles elimination goals are achieved throughout the world.
